# Partial lateral patellar facetectomy in primary total knee arthroplasty: a common addition with limited support

**DOI:** 10.1530/EOR-2025-0163

**Published:** 2026-03-02

**Authors:** Clément Horteur, Andrea Salvi, Pierre Girard, Benoit Gaulin, Brice Rubens Duval, Clémentine Rieussec, Mina W Morcos

**Affiliations:** ^1^ Hôpital Maisonneuve-Rosemont, Montreal University, Surgery Department, Montréal, Québec, Canada; ^2^ Department of Orthopaedic Surgery and Sport Traumaotlogy, Grenoble Alpes University Grenoble South Teaching Hospital, Echirolles, France

**Keywords:** knee, total knee arthroplasty, patella

## Abstract

- Partial lateral patellar facetectomy (PLPF) is a surgical procedure that consists in removing a part of the lateral facet of the patella.
- It has been first described as a surgical treatment for isolated external patellofemoral osteoarthritis. Following the same biomechanical effects, some authors proposed to perform PLPF in primary total knee arthroplasty to enhance patellar tracking and reduce the risk of anterior knee pain, whether the patella resurfaced or not.
- According to few studies of low level of evidence, functional scores are not improved when performing systematic PLPF.
- Current data are controversial regarding the role of systematic PLPF in enhancing patellar tracking.
- No evidence exists that PLPF protects un-resurfaced patella from revision for PF issues after TKA.
- In light of the available literature, PLPF cannot be recommended systematically in primary or revision TKA. However, precise relevant indications can be proposed.

## Introduction

Partial lateral patellar facetectomy (PLPF) is a surgical procedure consisting of removing a part of the patella’s lateral facet. It was first described by O’Donoghe (1) in 1972 as a surgical treatment for isolated external patellofemoral (PF) osteoarthritis (OA), also known as lateral facet syndrome (2). Recent studies reported good outcomes for this procedure performed open (3) or arthroscopically assisted (4). Pain relief is achieved through various methods that complement each other to reduce lateral PF pressure. Moreover, PLPF eliminates exposed patellar subchondral bone, which can potentially cause pain when in contact with the lateral trochlear groove. These effects, among others, have led some surgeons to add PLPF when performing primary total knee arthroplasty (TKA), regardless of whether the patella is resurfaced or not (5, 6, 7, 8, 9, 10). The management of the patellofemoral joint during TKA has always been an issue for knee surgeons since anterior knee pain is described as one of the most common causes of dissatisfaction after TKA (11). Various factors, depending on implant characteristics and positioning, affect patellofemoral kinematics (12). Any change from physiological patellofemoral anatomy and biomechanics may increase patellofemoral contact pressure and stress, thus potentially generating pain.

A functional increase in PF contact pressure can be consecutive to the decreased lever arm of the extensor mechanism (patellofemoral under-stuffing or paradoxical motion), which causes quadriceps muscle overuse and an increase in generation forces on the patella. On the other hand, direct mechanical consequences of TKA implantation may cause anterior knee pain due to augmented lateral PF retinaculum (LPFR) tension and PF contact pressure. PLPF can possibly prevent the latter as a safer option than lateral retinaculum release (13). For some surgeons, PLPF is indicated in TKA to enhance patellar tracking (10) and reduce the risk of patellar lateral facet impingement (LFI) with the lateral edge of the prosthetic trochlear groove (14). In 2000, Wachtl and Jakob (15) were the first to propose PLPF in TKA, reporting 85% good radiological patellar tracking in a series of 76 patients operated on in the late nineties.

Despite its widespread use in routine knee replacement, precise definition of PLPF in TKA is debated, and its efficacy in enhancing functional outcomes and patellar tracking remains unclear. This review aims to point out the biomechanical rationale of PLPF during TKA, describe the surgical technique and report previously published data on functional outcomes, patellar tracking, complications and patellar survivorship whether PLPF is performed or not during TKA.

### Anatomical and biomechanical considerations

#### Anatomy

The lateral aspect of the PF joint consists of the lateral articular facet of the patella, the lateral trochlear groove, the lateral capsule, ligaments and the retinaculum. PF joint function and stability are closely dependent on the integrity and congruency of its osteocartilaginous structures, dynamic muscular stabilizers and lateral static soft tissues, which counterbalance the medial-sided forces (16). In the normal knee, PF contact pressures during weight-bearing are usually scattered between medial and lateral facets throughout the range of motion (17). During physical activity, peak contact pressure might be augmented on the lateral side of the PF joint, as reported in the finite element assessment of strong eccentric quadriceps contraction (18). Pathologic PF joint functioning (PF instability, lower limb malalignment, dysplasia, malrotation, etc.) leads mostly to increased constraints on the lateral facet, as demonstrated by the strong predominance of lateral facet involvement over medial among cases of PF OA (19).

**Figure 1:**
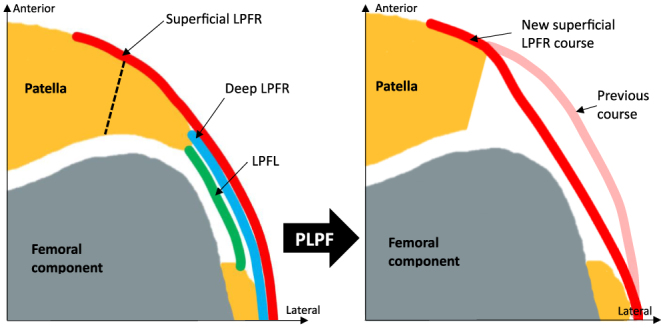
Illustration of the ‘tenting effect’ reduction and partial lateral patellofemoral retinaculum (LPFR) release after PLPF.

The lateral PF soft tissue can be described as a three-layer complex (Fig. 1). The superficial layer of the LPFR is composed of fibers from the quadriceps muscle aponeurosis and the iliotibial band (ITB). These fibers merge and work as a mixed statistic and dynamic stabilizing structure inserted on the proximal and anterior part of the patella laterally. The deep layer of the LPFR consists of the ITB’s deep transverse fibers and fibers originating from the vastus lateralis tendon. Its attachment is located on the lateral edge of the patella. Finally, the capsular layer runs beneath the LPFR, with condensations forming the patellofemoral, patellomeniscal and patellotibial ligaments. The lateral patellofemoral ligament (LPFL) is the thickest recognizable structure anchored to the lateral patella at its widest part and runs posteriorly to its femoral attachment on the lateral epicondyle (16, 20).

Histologic analysis of the LPFR shows that this structure has the highest incidence of sensitive nerve endings in the soft tissues around the knee, making it prone to generating pain when overstretched (21).

Finally, the only vascular structure notable is the lateral superior genicular artery. It runs from posterior to anterior between the capsule and the deep layer of the LPFR. The artery then courses more proximally toward the proximal edge of the patella (20). As a result, contrary to LPFR release, the lateral superior genicular artery is not at risk of damage when performing PLPF.

#### Causes of LPFR stretching and increased lateral PF contact pressure after TKA

Direct mechanical consequences of TKA implantation can contribute to the occurrence of LPFR stretching and an increase in lateral PF contact pressure:

- Anterior overstuffing consecutive to excessive size or anteriorization of the femoral component, or insufficient patellar cut thickness.
- Lateral joint line distalization during mechanically aligned TKA, especially when correcting congenital valgus alignment using a medial surgical approach (22, 23).
- Medial rotation of the prosthetic trochlear groove compared to the native one requires LPFR stretching to allow acceptable patellar tracking. It can be secondary to medial malrotation of the femoral component. Hence, many authors recommend systematic external rotation of the femoral component (24). Furthermore, relative native trochlear groove orientation compared to the posterior condylar axis is variable in the population. Since standard components do not offer decoupling of the trochlear groove from the posterior condylar axis, setting the femoral rotation with a posterior referenced instrumentation may lead to trochlear groove malrotation in many patients (12).

Personalizing TKA implantation to respect the patient’s native anatomy (unrestricted or restricted kinematic alignment (KA) (25, 26)) appears as the cornerstone to avoid the above-mentioned causes of LPFR stretching and lateral PF increased contact pressure. However, unrestricted KA shows certain limitations in the case of preoperative PF unbalance, which may persist postoperatively (27, 28). Indeed, Dossett *et al.* (29) and Howell *et al.* (30) reported PF tilt to be the main cause of revision after unrestricted KA TKA. As an alternative, the functional alignment technique was introduced to address two aspects that were neglected or taken for granted by the unrestricted KA technique: the constitutional trochlear position and the wide natural variation of soft tissue laxity (31). Finally, the use of modern prosthesis design with ‘patella-friendly’ features should be the gold standard in implant choice to reduce PF issues after TKA (32).

#### Effect of PLPF on PF contact pressure and LPFR tension

PLPF may be beneficial in TKA by reducing non-physiological PF contact pressure in two different ways potentiating one another. First, a lateral retinacular decompression is achieved by eliminating the ‘tenting effect’ of the lateral edge of the patella and its osteophytes underneath the LPFR (15) (Fig. 1). Second, the insertions of the lateral patellofemoral (PF) ligament and the deep layer of the lateral retinaculum are released with the bony resection (33), offering partial lateral retinaculum release as its superficial layer remains attached to the anterior aspect of the patella when performed under an intracapsular approach (20) (Fig. 1). This may limit pain symptoms that can follow LPFR over-tension because of its histologic characteristics mentioned above. The effect of PLPF on PF contact pressure is supported by Yuenyongviwat *et al.* (13) in a biomechanical cadaveric study. The authors measured PF contact pressure, thanks to a pressure-sensitive sensor applied on the articular surface of the native patella after implanting TKA in eight knees from four fresh-frozen specimens. PF contact pressure was recorded while performing passive knee flexion from full extension to maximum flexion. Peak PF contact pressure dropped on average from 3.43 ± 0.66 newtons (N) to 1.88 ± 0.55 N and 1.22 ± 0.49 N after 10 and 20% of lateral facetectomy were successively performed, respectively.

#### Lateral facet impingement

**Figure 2:**
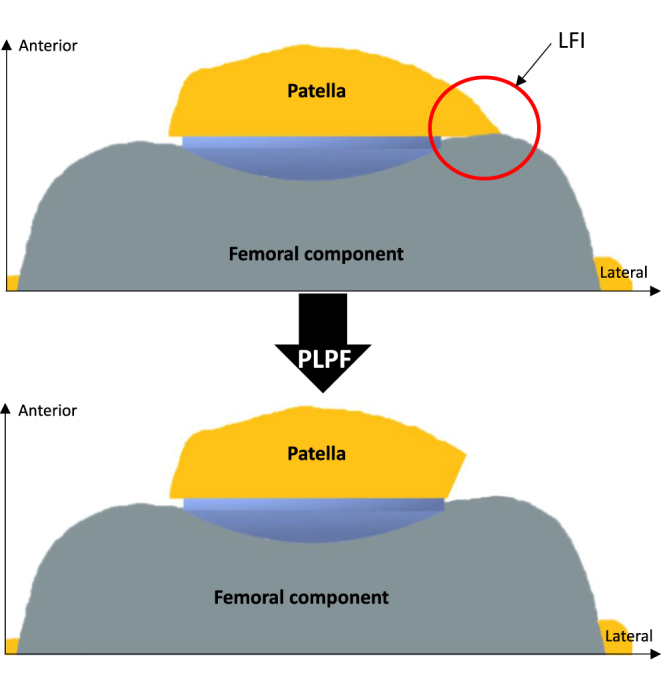
Illustration of LFI prevented by PLPF in resurfaced patella.

**Figure 3:**
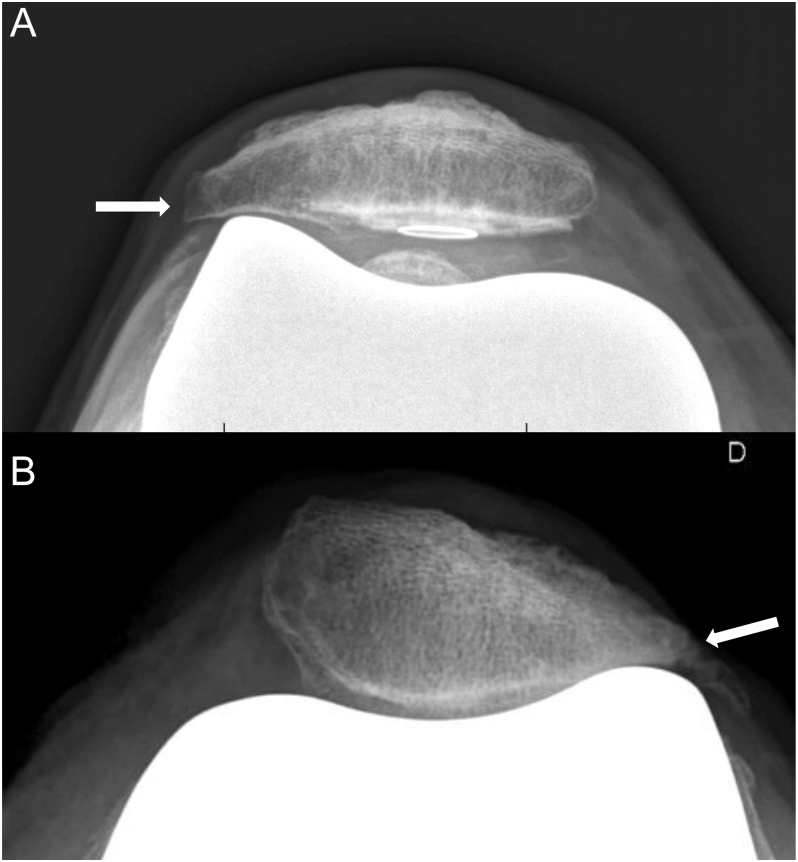
LFI consecutive to patellar button medialization and prominent lateral trochlear groove design (A). Lateral facet wear in non-resurfaced patella (B).

In 1995, Doerr & Eckhoff (34) introduced the concept of LFI in painful TKA with a resurfaced patella. It happens when insufficient coverage of the patella by its component allows abnormal contact between the lateral aspect of the patellar cut and the prosthetic femoral condyle (Fig. 2). It is favored by medialization, undersizing of the patellar button, the use of femoral implants showing prominent lateral trochlear groove design and the use of alignments other than mechanical alignment, where the femoral implants might not always be suited for. Careful LFI diagnosis includes precise lateral facet pain associated with typical radiologic findings (Fig. 3A) when other possible causes of painful TKA have been ruled out. Systematic resection of an uncovered lateral facet by the patellar component appears relevant to prevent LFI, especially when medializing the component, as recommended by many others to improve patellar tracking (35). However, the occurrence of symptomatic LFI remains rare, as only 0.3% of TKA revisions were motivated by LFI according to Nikolaus *et al.* (14) in a series of 3,361 revisions.

In non-resurfaced patella, PLPF eliminates the subchondral bone exposed laterally in the worn patella. Furthermore, it prevents abnormal contact between the lateral facet of the patella and the femoral component, especially when using an implant showing deep and narrow trochlear groove design, following the concept of LFI initially described in the resurfaced patella. However, altered PF kinematics or suboptimal patellar tracking after TKA can lead to lateral facet wear even with ‘patella-friendly’ implants (Fig. 3B). Finally, when severe wear renders the patella too thin to be safely resurfaced, PLPF can be performed to partially correct the abnormal concavity of the lateral facet.

### Surgical technique of PLPF during total knee arthroplasty

Partial lateral facetectomy can be performed at any step of the procedure. Usually done when trial implants are in place, it can also be performed as an early step to facilitate patella retraction and knee exposure when compromised by patellar deformation and osteophytes.

**Figure 4:**
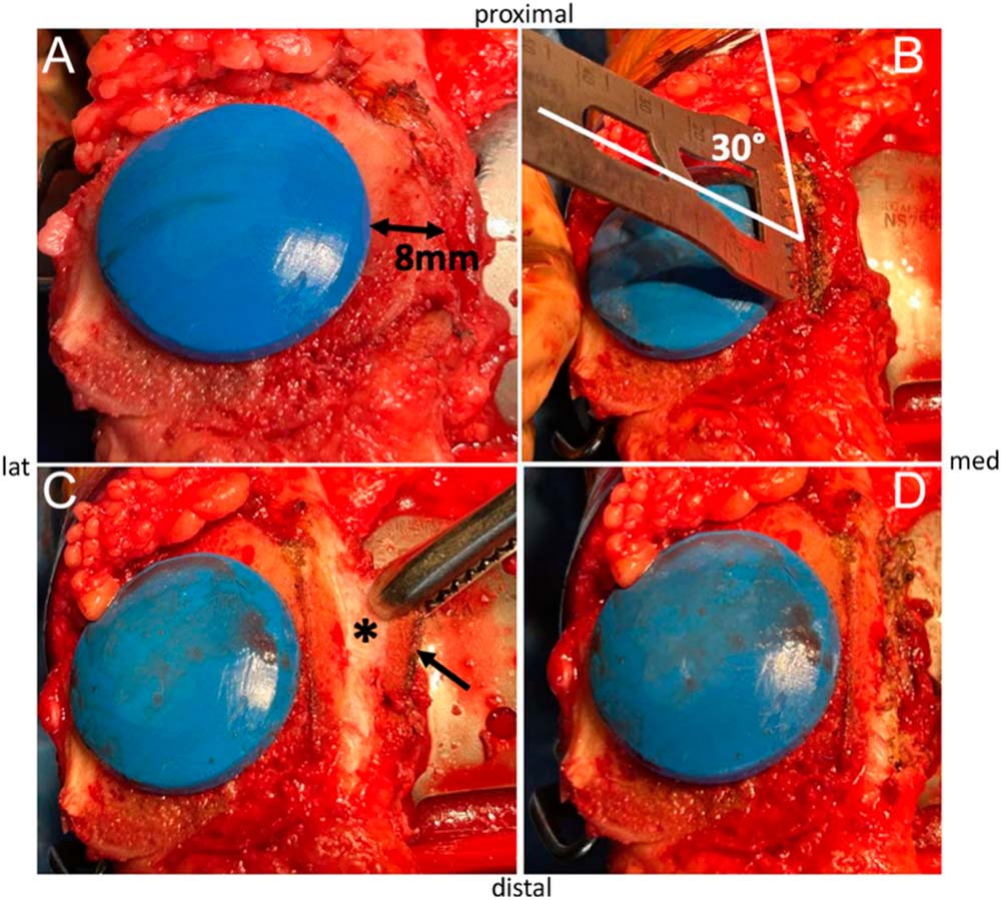
Surgical technique on resurfaced patella.

**Figure 5:**
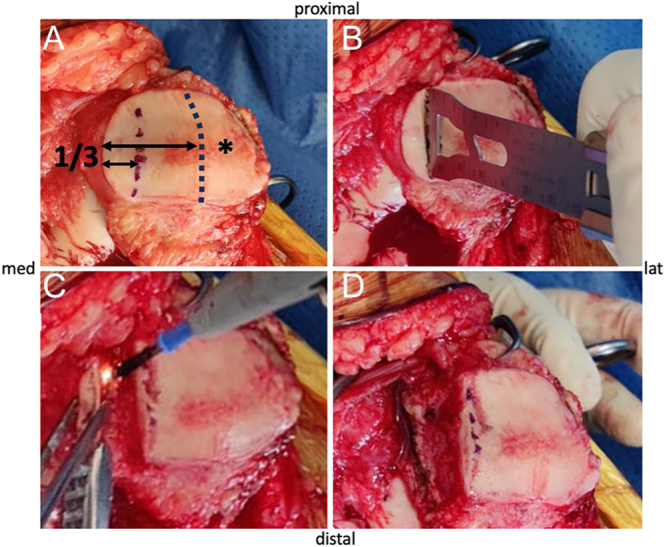
Surgical technique on non-resurfaced patella.

**Figure 6:**
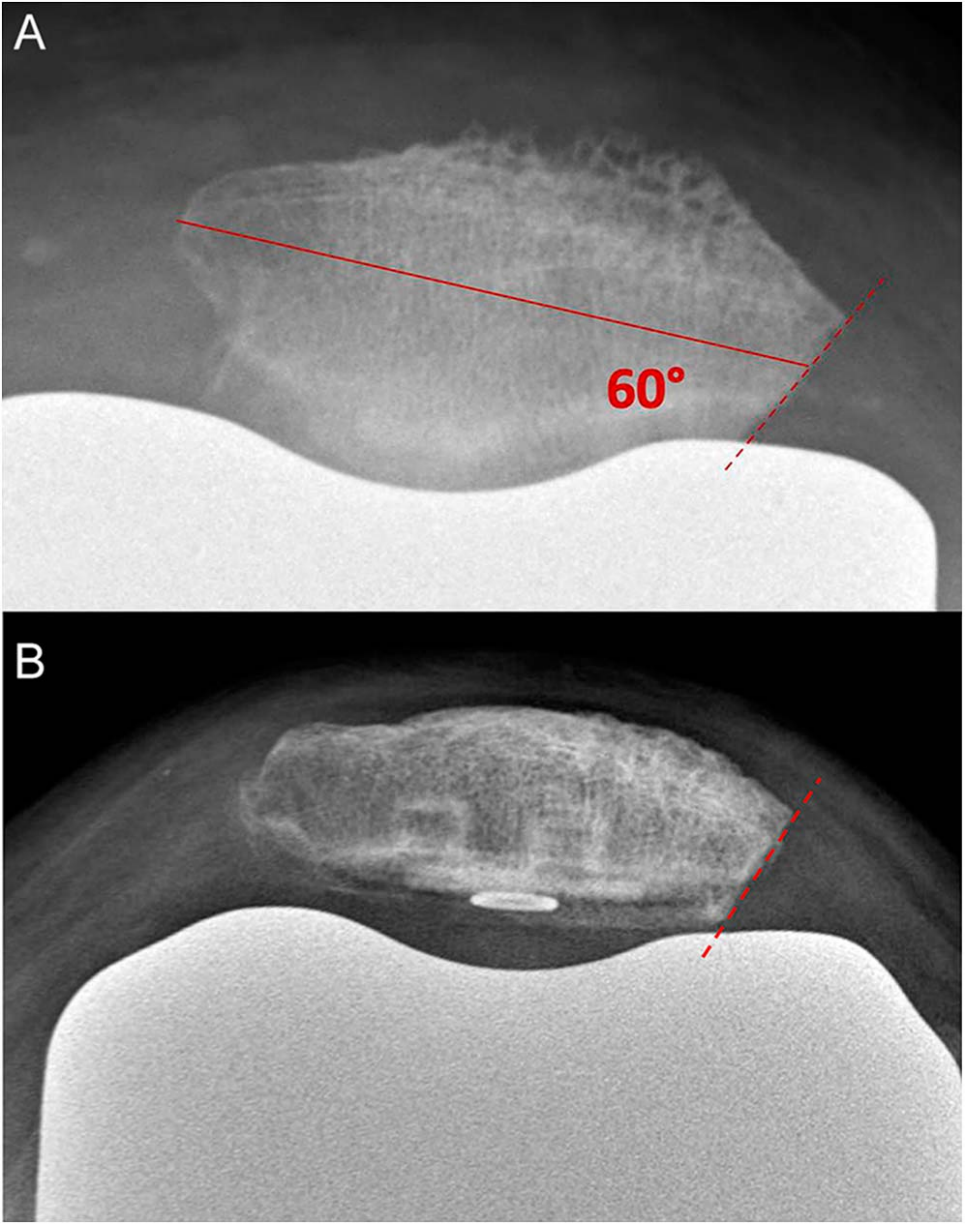
Postoperative radiographs after partial lateral facetectomy (skyline view). (A) Un-resurfaced patella; (B) resurfaced patella.

PLPF is carried out from an intracapsular approach. The knee is positioned in full extension before the patella is everted and stabilized with handheld surgical clamps. The osteotomy level can be marked with a demographic pen. In resurfaced patellae (Fig. 4), the osteotomy is done flush with the patellar component (5, 7) or 1–5 mm laterally from the implant (6). In non-resurfaced patellae (Fig. 5), the width of the resection remains controversial, ranging from 6 to 20 mm (5, 8, 15). On average, a 10 mm resection can be recommended and adapted to the size of the patella, which corroborates the definition of Lakstein *et al.* (10), who proposed to resect a third of the lateral facet. The facetectomy is performed with an oscillating saw and oriented in the sagittal plane, with possible lateral angulation up to 45° (Fig. 6). The more sagittal the osteotomy plane, the greater the retinacular decompression achieved, at the cost of a higher risk of superficial LPFR damage during its release from the bony fragment. The use of an osteotome appears unsuitable because of high subchondral bone density, insufficient stability of the everted patella and risk of uncontrolled iatrogenic fracture. Subperiosteal dissection of the superficial LPFR is carried out using an electric cautery or a blade, pulling the bony fragment upward to stretch and expose the deep LPFR. The bony fragment is then detached with caution to preserve the superficial LPFR. Eventually, sharp edges at the proximal and distal aspect of the osteotomy can be shaped with the saw.

### Results on clinical outcomes

To our knowledge, eight studies reported outcomes of PLPF during TKA on both resurfaced and non-resurfaced patella. Of the eight, two were case series (15, 36) and six were comparative studies with a control group in which no PLPF were performed (5, 6, 7, 8, 9, 10). Only retrospective (level of evidence III) studies have been published to date, reporting data on small cohorts. As mentioned by the authors, nearly all patients included in these studies underwent TKA using a medial parapatellar approach, posterior-stabilized implants and mechanical alignment technique.

Lakstein *et al.* (10) compared 23 patients with PLPF and 46 patients with LPFR release at a 3-year follow-up. For all patients, patellar tracking was deemed unsatisfactory at the conclusion of prosthesis implantation. The authors reported similar patient-reported outcome measures (PROMs) according to the Knee Society Score (KSS) and Feller score (assessing patellofemoral function) between both groups. Moghtadaei *et al.* (9) reached the same conclusions at a 2.5-year follow-up regarding functional score (KSS) when comparing simple patellar osteophyte removal to PLPF in 24 and 22 patients, respectively. Of the four other studies, two of them (6, 8) reported a significant improvement in functional knee and patellofemoral scores, while the other two reported comparable outcomes between the groups. None of these studies except one (8) reported the comparability of preoperative PF OA state and history of PF instability between groups. Patellar denervation was comparable between groups in two studies (5, 9) and unspecified in the others. Significant differences in functional scores were not preferentially reported in either resurfaced patella or patella-retaining series.

Possible complications when performing PLPF may include a complete unintended superficial LPFR section, injury to the superolateral genicular artery, and, consequently, subcutaneous hematoma or long-term avascular necrosis of the patella. Despite the difficulty of identifying PLPF as a cause of these multifactorial complications, the procedure can be considered safe since no complications directly related to the adjunction of PLPF during TKA have been reported to date. Finally, the patellar bone cut and its related residual bone debris may theoretically contribute to ossifications in the LPFR; however, no such cases or pain associations have been reported.

Still, based on the current literature, there is no clear evidence that systematic PLPF in TKA improves functional outcomes. Most of the studies published do not report PROM improvement when performing PLPF in TKA. Furthermore, there is a lack of high-level evidence study.

### Results on patellar tracking

Patellar tracking can be objectively assessed on Merchant view radiographs by measuring lateral translation and tilt of the patella (37, 38). Three comparative studies can be highlighted. Lakstein *et al.* (10) reported a similar lateral tilt (<2°) and translation (<2 mm) in a PLPF and a LPFR release group and a single outlier >8° in the LPFR release group. The authors considered PLPF as an efficient alternative to LPFR release in enhancing patellar tracking. Nagashima *et al.* (7) did not notice statistical differences in patellar lateral tilt (0° versus 0.4° on average, *P* = 0.42) and translation (2.4 versus 2.5 mm on average, *P* = 0.92) after TKA between 47 patients with PLPF and 37 patients without. Conversely, Kim *et al.* (5) reported a significant improvement in patellar tracking on both resurfaced and non-resurfaced patella. On resurfaced patella, patellar lateral tilt and translation were reduced by 1.6° (*P* = 0.02) and 1.3 mm (*P* = 0.06), respectively. Results were even more significant on non-resurfaced patella, showing a decrease in patellar lateral tilt and translation by 3° (*P* < 0.001) and 1.7 mm (*P* = 0.004), respectively.

Current data are controversial regarding the role of PLPF in enhancing patellar tracking. The level of evidence remains insufficient as none of these studies reported comparability between groups in terms of risk factors for postoperative patellar maltracking: neither preoperative factors (PF osteoarthritis, PF instability and lower limb alignment), nor intraoperative ones (implant positioning). PLPF could be suitable when a slight patellar tilt is noted during an intraoperative PF stability test, but certainly not in cases of patella subluxation, which require verification of correct implant positioning before considering extensive LPFR release.

### Results on patellar survivorship

Only one study assessed the relationship between PLPF during primary TKA and the survivorship of an un-resurfaced or native patella. Gunderson *et al.* (39) compared patellar survivorship at an 8.5-year follow-up between 823 patella-retaining TKA with PLPF and 230 without it. The overall revision-free survivorship was 98.9%, with no difference between groups. One case of patellar revision was observed in the PLPF group, while three cases were reported in the other group. This difference turned out to be statistically significant (0.1 versus 1.3%, *P* = 0.035). However, among the three cases in the no-PLPF group, two patellar revisions were indicated for global instability and one for aseptic loosening, and thus not related to PF issues but rather performed in the setting of femoral and tibial component revision. Moreover, the only revision in the PLPF group was prompted by unexplained knee pain. Thus, no evidence exists that PLPF protects un-resurfaced patella from revision for PF issues after TKA.

### General considerations for knee arthroplasty revision for patellofemoral issues

PLPF in TKA was also described as a patellar revision procedure for anterior knee pain. Pagenstert *et al.* (40) compared PLPF and patellar resurfacing procedures for treating anterior knee pain (lateral facet syndrome) after un-resurfaced patella TKA. They reported better outcomes in patients undergoing PLPF associated with medial reefing than in a matched group treated by patellar resurfacing. The study included 34 patients per group, for which preoperative PF score (Kujala score) were comparable (43.9 versus 42.9, *P* = 0.708), while the same score at a 3.4-year follow-up was 72.4 in the resurfacing group and 79.4 in the PLPF group (*P* = 0.007). The authors also highlighted the importance of eliminating any other possible cause of painful TKA by performing preoperative investigations, including at least synovial fluid analysis to rule out infection and CT-based implant positioning assessment.

In the case of symptomatic LFI after a resurfaced patella, Cercek *et al.* (41) suggested surgical treatment consisting of PLPF associated or not with a revision of the patellar component. Revision of the patellar component was only considered when the previous implant was deemed dramatically undersized. They reported good outcomes in 18 patients, while Nikolaus *et al.* (14) drew more cautious conclusions, mentioning a moderate functional score improvement, and only two-thirds of patients satisfied in a case series of 11 patients.

No study reported the effect of PLPF during TKA revision on postoperative patellar tracking.

### Summary of surgical indications

In light of the available literature, PLPF cannot be recommended systematically in primary or revision TKA. However, precise relevant indications can be proposed. Among them, some require further research to be fully supported (Table 1).

**Table 1:** PLPF indications during TKA.

TKA (primary and revision)	
Un-resurfaced patella	Resurfaced patella
Lateral facet OA	>5 mm of uncovered lateral facet
Severe patella wear (concave deformation) making the patella too thin to be resurfaced	Diagnosis of LFI*
Deep and narrow trochlear groove design	
Patellar dysplasia (Wiberg type III)	
Slightly enhance patellar tracking	

PLPF, partial lateral patellar facetectomy; TKA, total knee arthroplasty; OA, osteoarthritis; LFI, lateral facet impingement.

* Only applicable to revision TKA.

## Conclusion

PLPF during primary TKA is a relevant, quick and safe procedure that can be performed in numerous indications to prevent postoperative PF symptoms in both resurfaced and non-resurfaced patella. Yet, there is a lack of evidence to recommend systematic PLPF during TKA, which should rather be considered in delineated indications.

## ICMJE Statement of Interest

The authors declare that there is no conflict of interest that could be perceived as prejudicing the impartiality of the work reported.

## Funding Statement

The Maisonneuve-Rosemont Foundation and the French Society for Orthopedic and Trauma Surgery (SOFCOT) funded this research, supporting the arthroplasty fellowship program.
